# 

**Published:** 1850-01

**Authors:** 


### CONTENTS:

### PART I.—ORIGINAL COMMUNICATIONS.

| | PAGE. |
|---|---|
| ART. 1. Case of Hypertrophy of the Heart with Valvular disease. By Samuel Thompson, M. D. | 372 |
| ART. 2. Essay on Trembles or Animal Poison· By J. M. Logan, M.D. | 380 |
| ART. 3. On the use of the Ethereal Solution of Gun Cotton, as an External Application in Erysipelas. By J. W. Freer, M.D. | 386 |
| ART. 4. Nitrate of Silver in Sub-Acute Dysentery. By Wm. Matthews, M.D., | 388 |
| ART. 5 Preparation of Mercurial Ointment. By Charles Brackett, M.D., | 390 |
| ART. 6. Asthma cured by external application. By Dr. Wm Townsend, | 391 |
| ART, 7, Tartar Emetic, Quinine and Morphine in Pneumonia of Miasmatic Districts. By E, C, Banks, M, D, | 392 |

### PART II.—REVIEWS AND NOTICES OF NEW WORKS.

| | |
|---|---|
| ART. 1. Parturition and the Principles and Practice of Obstetrics. By W. Tyler Smith, M.D., &c., | 400 |
| ART. 2. Lectures on the Diseases of Infancy and childhood. By Chas. West, M.D., F. R. S. | 402 |
| ART. 3. Remarks on the Obstetrical Forceps, with a description of an Instrument employed. By J. P. White, M.D., | 404 |
| ART. 4. Contributions to Physiology. By Bennet Dowler, M.D., &c., | 405 |
| ART. 5. A Treatise on Diseases of the Bones. By Edward Stanley. F. R. S., &c. | 410 |

### PART III.—SELECTIONS.

| | |
|---|---|
| ART. 1. Animalculæ in the Atmosphere of Cholera Patients, By R. D, Mussey, M. D. &c., | 411 |
| ART. 2. On the Use and Administration of Cod-Liver Oil in Pulmonary Consumption—By J. C. B. Williams, M.D., | 415 |
| ART. 3. Cryptogamous Origin of Cholera, | 426 |
| ART. 4. Treatment of Hemorrhoids with Nitric Acid—By S. Weed, M.D. | 435 |
| ART. 5. Compression of the Aorta in Uterine Hemorrhage—By Robt. Crane, M.D. | 438 |

### PART IV.—EDITORIAL.

| | |
|---|---|
| ART. 1. Free Medical Schools, | 440 |
| ART. 2. Proceedings of the Æsculapean Society, | 449 |
| ART. 3. Official Call for a State Medical Convention, | 455 |
| ART. 4. Illinois State Medical Convention, | 456 |
| ART. 5. Appointment of Demonstrator of Anatomy in Rush Medical College, | 457 |
| ART. 6. The Concours, | 458 |
| ART. 7. Dr. Latta's Circular, | 459 |
| ART. 8. Obituary, | 461 |
| ART. 9. Medical Miscellany, | 461 |

